# Suspected paracetamol overdose in Monrovia, Liberia: a matched case–control study

**DOI:** 10.1186/s12887-020-2008-3

**Published:** 2020-03-30

**Authors:** Mohamad K. Haidar, Florian Vogt, Kensuke Takahashi, Fanny Henaff, Lisa Umphrey, Nikola Morton, Luke Bawo, Joseph Kerkula, Robin Ferner, Klaudia Porten, Frederic J. Baud

**Affiliations:** 1 Epicentre, 8 Rue Saint Sabin, 75011 Paris, France; 2 UMR 8257, Université Paris Descartes, Paris, France; 3 Médecins sans Frontières – Operational Center Paris, Paris, France; 4 Ministry of Health and Social Welfare, Monrovia, Liberia; 5 Institute of Clinical Sciences, University of Birmingham, Birmingham, England; 6 Assistance Publique – Hôpitaux de Paris, Paris, France; 7 University Paris Diderot, Paris, France; 8 EA7323, Evaluation of prenatal and paediatric therapeutics and pharmacology, Université Paris Descartes, Paris, France

**Keywords:** Paracetamol, Liberia, Paediatrics, Liver insufficiency

## Abstract

**Background:**

A cluster of cases of unexplained multi-organ failure was reported in children at Bardnesville Junction Hospital (BJH), Monrovia, Liberia. Prior to admission, children’s caregivers reported antibiotic, antimalarial, paracetamol, and traditional treatment consumption. Since we could not exclude a toxic aetiology, and paracetamol overdose in particular, we implemented prospective syndromic surveillance to better define the clinical characteristics of these children. To investigate risk factors, we performed a case–control study.

**Methods:**

The investigation was conducted in BJH between July 2015 and January 2016. In-hospital syndromic surveillance identified children with at least two of the following symptoms: respiratory distress with normal pulse oximetry while breathing ambient air; altered consciousness; hypoglycaemia; jaundice; and hepatomegaly. After refining the case definition to better reflect potential risk factors for hepatic dysfunction, we selected cases identified from syndromic surveillance for a matched case–control study. Cases were matched with in-hospital and community-based controls by age, sex, month of illness/admission, severity (in-hospital), and proximity of residence (community).

**Results:**

Between July and December 2015, 77 case-patients were captured by syndromic surveillance; 68 (88%) were under three years old and 35 (46%) died during hospitalisation. Of these 77, 30 children met our case definition and were matched with 53 hospital and 48 community controls. Paracetamol was the most frequently reported medication taken by the cases and both control groups. The odds of caregivers reporting supra-therapeutic paracetamol consumption prior to admission was higher in cases compared to controls (OR 6.6, 95% CI 2.1–21.3). Plasma paracetamol concentration on day of admission was available for 19 cases and exceeded 10 μg/mL in 10/13 samples collected on day one of admission, and 4/9 (44%) collected on day two.

**Conclusions:**

In a context with limited diagnostic capacity, this study highlights the possibility of supratherapeutic doses of paracetamol as a factor in multi-organ failure in a cohort of children admitted to BJH. In this setting, a careful history of pre-admission paracetamol consumption may alert clinicians to the possibility of overdose, even when confirmatory laboratory analysis is unavailable. Further studies may help define additional toxicological characteristics in such contexts to improve diagnoses.

## Background

Used widely in children as an analgesic and antipyretic medication, paracetamol is found in over-the-counter and prescription products worldwide [1]. Paracetamol has a well-established safety profile when recommended doses are administered [2]. Overdose may occur after a single ingestion of a large amount of paracetamol or paracetamol-containing medication, or repeated ingestion of smaller amounts that eventually exceed the recommended cumulative dosage. Both prolonged use and acute overdose may cause hepatic injury [3]. Although reported rates vary globally, paracetamol is considered the most common agent consumed in overdose particularly among children aged less than 6 years [4, 5]. Various studies have also reported acute liver failure (ALF) due to single-dose overdose or repeated exposure to supratherapeutic doses of paracetamol [6].

Data on paracetamol overdose from Sub-Saharan Africa is limited [7]. Poor understanding of paediatric dosing has resulted in reported overdose in Nigeria and South Africa [8, 9]. A community survey in Nigeria reported 28% of children under 5 years had ingested supratherapeutic doses of paracetamol in tablet form in the previous month [10]. In South-Africa, paracetamol was identified as the second most frequent presumed toxic cause of hospitalisation among children [11]. Further compounding the toxic effects are possible co-administration of other toxins in addition to paracetamol, for example diethylene glycol mixed with paracetamol in Nigeria manufactured locally [12].

In addition to conventional medication, many commonly-used traditional remedies have been associated with hepatotoxicity [13]. Studies have identified herbs and fungi commonly used as traditional remedies which may cause acute or chronic liver injury [14, 15]. In sub-Saharan Africa, consumption of traditional herbal treatments, is known to be common practice and in some cases associated with toxicity [16].

Between April 1 and May 30, 2015, 10 children admitted to Bardnesville Junction Hospital (BJH) in Monrovia, Liberia, suffered signs of multi-organ failure and hepatotoxicity comprising 1.3% (10/748) of total admissions during the same period. Prior to admission, children’s parents reported antibiotic, antimalarial, paracetamol, and traditional treatment consumption. Since a toxic aetiology, paracetamol overdose in particular, could not be ruled out, we implemented prospective syndromic surveillance to better define the clinical characteristics of these children. To investigate risk factors, we performed a case–control study using a case definition refined during syndromic surveillance.

## Methods

### Study setting

The population of Liberia is estimated to be 4.5 million people [17], 25% of whom are under 5 years old [18]. Montserrado County is home to one-third of Liberia’s population and the capital Monrovia [19]. The most recent Liberian Demographic Health Survey in 2013 reports malaria (29%), diarrhoea of any cause (20%), acute respiratory infections (9%), and severe acute malnutrition (2%) among the most common reported morbidities in children under five in the county [18].

Opened in April 2015, BJH is a paediatric referral hospital in Monrovia. The 74-bed hospital was opened during the West African Ebola outbreak (2014–2016) to provide secondary care to non-Ebola paediatric patients. During the outbreak, essential primary healthcare services decreased substantially with estimates suggesting malaria and other infectious diseases increased during this time [20].

### Syndromic surveillance

Children admitted to BJH between July and December 2015 and aged one month to five years were screened on admission Children with at least two of the following symptoms were considered case-patients: respiratory distress with normal pulse oximetry (saturation > 94% while breathing ambient air); altered consciousness; hypoglycaemia; jaundice; or hepatomegaly.

A database of all children meeting this definition was created, and included data on vital signs and clinical symptoms on admission and 24 h after, laboratory tests if available on admission and anytime during hospitalisation, treatment given in the hospital, and hospitalisation outcome.

### Case-control study

#### Case definition

To better reflect potential risk factors for hepatic dysfunction possibly linked to paracetamol overdose, cases were exhaustively selected among those case-patients identified by syndromic surveillance between September and December 2015. To be considered as a case in the case-control study, respiratory distress (tachypnoea, bradypnoea, nasal flaring, grunting, inter-, or sub-costal retractions) and normal pulse oximetry (SpO_2_ > 94% while breathing ambient air) were necessary; along with at least one of the following: hepatomegaly, hypoglycaemia, or absence of fever at admission (defined as < 38 °C).

#### Definition of controls

Hospital-based controls were children aged one month to five years admitted to BJH who did not meet the definition of a case. Two hospital-based controls were matched to each case by age group (< 1 year, 1–3 years, and 3–5 years), sex, severity based on emergency room triage, the paediatric early warning signs (PEWS) score [21], and calendar month of admission. We matched by severity to reduce the possibility of reverse causality, as sicker children might have consumed more of the toxic agent prior to hospitalisation if a toxic agent was identified. Similarly, matching by calendar month of admission was to control for any seasonal factor, such as malaria, affecting exposure to a potential putative toxic agent or infection.

A second set of controls was selected from the community, eligible if they reported one episode of non-traumatic illness that did not result in admission to hospital. Two community-based controls were matched to each case by age group, sex, month of illness, and proximity of residence. We matched by proximity of residence to control for any local potential exposure within a community or an area of Monrovia. Community-based controls were selected starting with the house closest to the case’s residence. House-to-house visits continued in a spiral until two controls within the same community were identified. Age of the child and the calendar month of illness were reported by the caretaker. Controls were selected between September 2015 and January 2016. A standardized face-to-face interview was conducted by trained study staff, addressing symptoms occurring and treatments consumed prior to hospitalisation.

#### Ascertainment of confounding factors

Vital signs and serum biochemistry laboratory tests (electrolytes, liver transaminase, total bilirubin, blood urea nitrogen and blood sugar) were defined according to standard reference ranges [22–24]. Hypoglycaemia was defined as blood glucose less than 60 mg/dL using a glucometer and screened upon admission. Estimated serum anion gap was calculated as per the formula [(Na^+^ + K^+^) – (Cl^−^ + Total CO_2_)]. Severe acute malnutrition was defined by either the presence of bilateral oedema, mid-upper arm circumference (MUAC) < 115 mm, or weight-for-height z-score < − 3 [25]. It was not possible to weigh community-based controls, so we used the mean weight-for-age (z-score) according to the World Health Organization standards [26]. All medicines consumed prior to hospitalisation were documented, as self-reported by the caretakers. Answers were recorded at the time of admission for the cases and hospital controls, or at the time of survey for community controls.

Study nurses sought medication dosage data by discussing medication history and showing samples of medications to caretakers. Nurses had samples of different common formulations of paracetamol and showed them to caretakers, who then indicated how many pills were given over a given period. For paracetamol, a reported supratherapeutic dose was considered to be more than an estimated 150 mg/kg body weight within 24 h or 75 mg/kg within 48 h [6, 27]. Among commonly-used antimalarials with known hepatotoxicity, we considered doses supratherapeutic if a child was: i) less than 12 months and reported consumption of more than 25 mg artesunate and 67.5 mg of amodiaquine for 3 days of treatment; or ii) older than 12 months and reported consuming more than 50 mg artesunate and 135 mg of amodiaquine for 3 days of treatment [28]. Other conventional hepatotoxic medications were rarely reported having been consumed by cases or controls; hence, we did not define their supratherapeutic doses.

Paracetamol intoxication was defined as a plasma paracetamol concentration of ≥10 μg/mL coupled with serum aspartate aminotransferase (AST) and/or alanine aminotransferase (ALT) more than twice the upper limit of normal (ULN), as suggested by Kozer et al. [27]. We used the plasma paracetamol concentration obtained on admission or the day after. As the investigation was conducted during the 2014–16 West African Ebola outbreak, patients were screened for signs of Ebola prior to admission. Post-mortem blood swabs were used to perform Ebola PCR testing in children who died during hospitalisation.

Plasma paracetamol concentrations do not correlate with the likelihood of increased serum transaminase activity in chronic exposure toxicity [29]. Therefore, the Rumack-Matthew nomogram, based on the pharmacokinetics of acute ingestions, does not enable predicting liver injury or hepatotoxicity in children with chronic exposure [29]. In fact, RSTI intoxication is similar to late presentations of acute poisoning in a way that plasma paracetamol concentration might become undetectable and difficult to interpret [27]. Hence, clinical approach always considers detectable and even quantifiable plasma paracetamol concentrations as suggestive of the presence of liver injury [27].

#### Toxicological laboratory procedures

We did not collect specific blood samples for toxicological analyses from children identified in the syndromic surveillance. For the case-control study, twenty-four plasma samples (residual amounts from planned blood draws used for treatment purposes) were available from 24 of the 30 children included as cases. No residual plasma samples were analysed from controls. Toxicological laboratories were unavailable in Liberia. Therefore, samples were stored at -20 °C before being transported for toxicological testing (Laboratoire de Toxicologie, Hôpital Raymond Poincaré, Garches, France). Liquid chromatography with high resolution mass spectrometry detection was used to quantify concentrations of paracetamol and other hepatotoxic drugs.

#### Statistical analyses

We assumed 80% exposure to the putative toxic agent consumed prior to hospitalisation among cases and 50% exposure among controls. These assumptions were based on an assumed odds ratio of four given prior estimates reported in the literature. We estimated a sample size of 30 cases and 60 controls provided 80% power to show this difference with a type I error of 5%. Vital signs, symptoms, and biochemical indices are presented as proportions, means, medians and ranges. Associations between the presence of paracetamol toxicity and potential risk factors were evaluated using odds ratios (ORs), *p*-values, and 95% confidence intervals (CI) derived from conditional stepwise logistic regression. Variables with *p* < 0.2 were included in the multivariable analysis. Univariate and multivariable analysis was conducted for each control group separately; however, to increase statistical power post-hoc we pooled the control groups. We considered two-sided tests to be statistically significant if *p* < 0.05. All statistical analyses were performed using STATA version 13.1 (College Station, Texas).

#### Ethical considerations

We obtained written informed consent for participation from the parents or guardians of participants enrolled in the case–control study. Parents’ or guardians’ of case-patients whose plasma samples were sent for toxicological analysis provided a separate written informed consent for sample storage and shipment. The study was approved by The Liberian National Research Ethical Board (reference: NREB-003-16).

## Results

Between July and December 2015, a total of 77 children between one month and five years who met the case definition were captured by the syndromic surveillance system, of whom 35 (46%) died during hospitalisation (Table 1). The most frequent signs of the syndrome were respiratory distress (99%; 84% without hypoxia); hepatomegaly (74%); tachycardia (68%), and altered consciousness (lethargy or coma) (77%). A quarter of the children had severe acute malnutrition, and 16 (21%) had a positive malaria rapid diagnostic test (RDT).

**Table 1:** Clinical and biochemical parameters of the case-patients included syndromic surveillance, July to December, 2015

	Total (***N*** = 77)		
	n (%)		n (%)
***Characteristics***		***Biochemical Indices***	
**Male**	40 (52)	**Hypoglycaemia**	
**Age**		Once	43 (57)
≤1 years	56 (73)	Continuous over 24 h	7 (9)
> 1–2 years	12 (15)	**AST level (*n*** **= 69)**	
> 2 years	9 (12)	< 2 times ULN	5 (7)
**Died**	35(46)	2–5 times the normal range	12 (17)
***Symptoms***		> 5 times the normal range	52 (75)
**Hyperthermia (total)**	43(56)	**AST activity > 1000 units/L**	35 (51)
Upon admission	26(34)	**ALT activity (n = 69)**	
2nd day of hospitalisation	5(6)	< 2 times the normal range	11 (16)
Continuous over 2 days	12(16)	2–5 times the normal range	11 (16)
**Respiratory distress (total)**	76 (99)	> 5 times the normal range	47 (68)
Upon admission	21 (27)	**ALT activity > 1000 units/L**	30 (44)
2nd day of hospitalisation	4 (5)	**ALP activity (n = 69)**	
Continued after 2 days post-admission	51 (66)	Normal	60 (87)
**Respiratory distress without Hypoxemia**	65 (84)	Mildly elevated < 2 times ULN	9 (13)
**Neurological Status**		**Total Bilirubin concentration (n = 69)**	
**Irritable (total)**	9 (12)	Normal	28 (41)
Upon admission	2 (3)	Mild Elevation	13 (19)
2nd day of hospitalisation (total)	6 (8)	Elevation of (2-3 mg/dl)	14 (20)
*Recovering*	5 (7)	Elevation of (> 3 mg/dl)	14 (20)
*Worsening*	1 (1)	**Serum Creatinine (*n*** **= 68)**	
Continuous over 2 days	1 (1)	Normal	7 (10)
**Lethargic (total)**	31 (40)	Elevation less than 1 mg/dl	32 (47)
Upon admission	11 (14)	Elevation (1–2 mg/dl)	16 (24)
2nd day of hospitalisation (total)	10 (13)	Elevation (> = 2 mg/dl)	13 (19)
*Recovering*	4 (5)	**Estimated Anion Gap > 18 mmol/L (*n*** **= 66)**	44 (67)
*Worsening*	6 (8)	**Elevated Blood Urea Nitrogen**	30 (43)
Continuous over 2 days	10 (13)	**Malaria (RDT)**	16 (21)
**Coma (total)**	28 (36)	**HIV positive (*n*** **= 45)**	3 (7)
Upon admission	10 (13)		
*Decreasing GCS*	8 (10)		
Continuous over 2 days	10 (13)		
**Hepatomegaly**	57 (74)		
**Tachycardia**	53 (68)		
Upon admission	24 (31)		
2nd day of hospitalisation	9 (12)		
Continued after 2 days of admission	20 (26)		
**Convulsions**	26 (34)		
**Severe Acute Malnutrition**	19 (25)		

From the 77 children identified by syndromic surveillance, 30 cases met our case definition and matched them with 53 hospital and 48 community controls. Thirty percent of the cases and 9.4% of controls died during hospitalisation (Table 2). Altered consciousness and convulsions were more frequent among the cases in comparison to the controls. Conversely, tachycardia was more frequent among the controls.

**Table 2:** Clinical profile and outcome of the cases and controls in the case-control study–univariate analysis

	Cases (***N*** = 30)	Hospital Controls (***N*** = 53)	
	N(%)	N(%)	Odds Ratio (95% CI)
**Symptoms and Biochemistry**			
Absence of Fever	15(50)	30(57)	0.8(0.4–1.7)
Hepatomegaly	27(90)	5(9)	
Respiratory Distress	30 (100)	36(68)	
Respiratory Distress with Hypoxemia	0)0)	11(21)	
Tachycardia	15(50)	77(76)	0.3(0.1–0.8)
Altered Consciousness	18(60)	12(23)	4.4(1.5–12.5)
Convulsions	14(47)	18(18)	3.7(1.5–9.2)
Diarrhoea	2(7)	11(20.8)	0.3(0.0–1.5)
Severe Acute Malnutrition	10(33)	17(32)	1.0(0.4–2.9)
Hypoglycaemia	24(80)	8(15)	
Malaria	2(7)	8(16)	
**Outcome of Hospitalisation**			
Discharged	18(60)	48(91)	
Transferred or discharged against medical advice	3(10)	0	
Deceased	9(30)	5(9)	12.9(2.8–58.9)

Among the 9/30 deceased, the mean time to death was 3 days of hospitalisation ranging from less than 24 h to 15 days. Among the 5/53 deceased controls, the mean time to death was 8 days ranging from 24 to 48 h to 11 days. Furthermore, less than half of the cases had normal saline or ringer lactate bolus administered during hospitalization. The majority, who received it, received only once upon admission. As tachycardia, high estimated anion gap, high creatinine and capillary refill are common with paracetamol toxicity in addition to shock, the lack of statistical association with fluid bolus usage strengthens the hypothesis of paracetamol surpatherapeutic poisoning ruling out cardiovascular shock.

Paracetamol was the most frequently reported conventional medication consumed by the cases and by both control groups. Boiled ‘Tonton leafs’was reported as the most frequently consumed, for both cases and controls, among traditional medications (Table 3). The odds of having reported paracetamol doses consistent with overdose, adjusted for potential hepatotoxins, was higher in cases compared with the controls (OR 6.6, 95% CI 2.1–21.3). In the matched case-control, 24 blood samples were available from cases, with 10 of these on the day of admission (Table 4). No samples were collected from controls and the five other samples were from children included in syndrome surveillance, but whom upon further examination did not meet the case definition (Table 4). A total of 9/10 (90%) cases had their samples collected on admission with plasma paracetamol concentrations exceeding 10 μg/mL, coinciding with elevated transaminase levels more than five times the ULN. Therapeutic concentrations of trimethoprim (15/24), sulfamethoxazole (15/24), and salicylic acid (8/24) were detected in the plasma of cases.

**Table 3:** Multivariate analysis of potential risk factors for the suspected toxic syndrome

	Cases (***N*** = 30)	Hospital Controls (N = 53)			***p***-value	Community Controls (***N*** = 48)			***p***-value	Pooled Controls (***N*** = 101)			
	n(%)	n(%)	OR (95% CI)	aOR^**a**^ (95%CI)		n(%)	OR(95% CI)	aOR* (95%CI)		n(%)	OR (95% CI)	aOR^**a**^ (95%CI)	***p***-value
**Paracetamol (total)**	26(87)	28(53)				46(96)				74(73)			
Paracetamol supratherapeutic dose	9(30)	5(9)	3.8 (1.1–12.4)	3.7 (1.0–13.1)	0.046	2(4)	13.6 (1.7–110.1)	13.6 (1.7–110.1)	0.014	7(7)	5.6 (1.9–16.9)	6.6 (2.1–21.3)	0.001
**Antibiotics**	16(53)	16(30)				42(88)				58(57)			
Co-trimoxazole	7(23)	3(6)	4.4 (1.0–17.1)	4.3 (1.0–18.6)	0.048	17(35)	0.6 (0.2–2.0)			20(20)	1.3 (0.5–3.3)		
Erythromycin	2(7)	0(0)				4(8)	0.9 (0.2–4.9)			4(4)	1.9 (0.3–10.4)		
**Anti-malaria Medication**	11(37)	12(23)				36(75)	–			48(48)			
Antimalarial drugs supratherapeutic doses	1(3)	0(0)				6(13)	0.3 (0.4–2.8)			6(6)	0.6 (0.1–5.3)		
**Traditional Medicine**	20(67)	18(34)				22(46)				40(40)			
“Tonton” Leaf	4(13)	4(8)	1.7 (0.4–6.8)			5(10)	1.4 (0.3–5.9)			9(9)	1.4 (0.4–5.3)		
“Monkey Apple” Leaf	3(10)	0(0)				4(8)	1.4 (0.3–6.1)			4(4)	2.4 (0.5–10.8)	3.4 (0.7–17.3)	0.148
“Wild Wild” Leaf	2(7)	3(6)	1.2 (0.2–7.0)			2(4)	2.0 (0.3–14.1)			5(5)	1.4(0.3–7.5)		
Pre-prepared traditional treatments	3(10)	1(2)	5.2 (0.5–50.4)	10.9 (0.7–160.9)	0.081	0(0)				1(1)	11.2 (1.2–108.1)		
“Lynch Street” Medicine	1(3)	2(4)	1 (0.0–11.0)			2(4)	0.6 (0.1–7.0)			4(4)	0.9 (0.1–8.0)		
Almond Bark	0(0)	0(0)				4(8)	–			4(4)			
Other traditional treatments	7(23)	10(19)	1.4 (0.5–4.6)			10(21)	0.9 (0.3–3.0)			20(20)	1.3 (0.5–3.5)		

^a^Adjusted odds ratios (aORs) are derived from stepwise conditional logistic regression models. The initial multivariate models included all the variables which were included in the univariate analysis presentedby the odds ratio (OR). Non-significant variables were eliminated to reach the final model as presented

**Table 4:** Paracetamol plasma concentrations in the case–control study

					None Detected	≤10 μg/mL	> 10 μg/mL
	N	Median (interquartile range) (μg/mL)	Mean (range) (μg/mL)	SD	n(%)	n(%)	n(%)
**Total**	24	11.6(1.4–41.0)	25.2(0–108.3)	33.2	4(17)	7(29)	13(54)
**Day of hospitalisation the sample was collected**							
Day One	10	20.3(12.1–56.3)	36.7(1.9–108.4)	36.7	0(0)	1(10)	9(90)
Day two	9	4.4(< 0.1–29.0)	25.3(0–105-.6)	36.8	2(22)	3(33)	4(44)
Day three	3	1.7(0–2.7)	1.5(0–2.7)	1.4	1(33)	2(67)	0(0)
More than 3 days	2	3.1(0–6.1)	3.1(0–6.1)	4.3	1(50)	1(50)	0(0)

## Discussion

We describe a cluster of severely ill children with high mortality in a reference hospital at the end of the West African Ebola epidemic. Although the clinical features described here are consistent with the possible time-course of toxic effects, and those of paracetamol in particular [30], the diversity of clinical presentations makes drawing conclusions difficult. The results of the case-control study should be interpreted as hypothesis-generating rather than confirmatory.

This study took place in a vulnerable paediatric population with high rates of malnutrition and malaria, during a critical time period during and following the Ebola epidemic. It is possible that children with severe acute malnutrition and malaria may be more susceptible to the hepatotoxic effects of paracetamol, and hence develop liver injury at lower levels of paracetamol ingestion than previously thought. Toxic effects similar to those we observed such as metabolic acidosis, hypoglycaemia, altered consciousness, are reported in delayed presentations of paracetamol-induced hepatotoxicity [31]. However, the magnitude of transaminase activity highlights a degree of liver injury not seen in either.

A definitive diagnosis of a toxic cause requires both analytical tests to detect a toxicant and the exclusion of non-toxic diagnoses. Plasma paracetamol concentration on admission remains the reference in the diagnosis of paracetamol poisoning. Our study has weaknesses both in limited measurements of plasma paracetamol concentrations and also in the exclusion of non-toxic causes, largely due to limited laboratory capacity in this setting. Nonetheless, this is common in many parts of sub-Saharan Africa, and we believe that these hypothesis-generating results, even with their limitations, are both new and important.

Among the cases with measured plasma paracetamol concentration, we note that there is debate over the most appropriate thresholds (10 or 20 μg/ml) [27, 32]. Among cases with a sample collected on the day of admission, almost all exceeded 10 μg/mL and concurrently had elevated transaminases consistent with paracetamol intoxication. Paracetamol concentrations exceeded 10 μg/mL in 4/9 samples on second day of hospitalisation.

Recently, measuring protein adducts was proposed as an experimental method to confirm paracetamol poisoning [33]. However, to our knowledge, this method is presently only of experimental value, not used routinely in diagnosing paracetamol intoxication anywhere in the world especially in low-resource settings [34].

Aside from toxic aetiologies, there are many other possible non-toxic diagnoses leading to hepatic insufficiency in children [35]. Malaria is a known cause of multiple organ dysfunction, including liver injury [36]. Sepsis, measles, yellow fever, Lassa fever, Ebola, and dengue fever may also cause liver injury. One possibility is that paracetamol was taken for a condition that itself caused liver toxicity. For example, transaminitis and hyperbilirubinemia may occur during viral infection [35], or malaria, whether or not paracetamol is concurrently taken. Transaminase activity obtained from Nigerian children with severe malaria was less than twice ULN with a mean of 139 IU/L AST and 73 IU/L ALT [37], significantly lower than in the children in our study, including those with malaria. Hepatitis E and A were not tested, however hepatitis E is known to cause less severe hepatitis in children [38]. Furthermore, children with confirmed hepatitis B and C were not included. Regarding traditional treatments, in this study none taken by patients were identified as toxic, nor did we find a statistical association with the putative syndrome. Nonetheless, this does not exclude the possibility of the ingestion of plants with toxic effects. Further, the combination of hepatotoxic drugs with paracetamol might have increased the risk of liver injury and hepatotoxicity.

The overall high mortality in all the groups included in this study precludes any comparison with what may occur in Western settings, especially with the presence various fatal conditions specific to Liberia.

### Limitations

There are several important limitations to this work. First, all assessments of conventional and traditional treatment consumption prior to admission relied upon report. The accuracy of self-reported dosage of medications consumed prior to hospitalisation among cases and hospital controls is unknown. Further, assumptions concerning supratherapeutic paracetamol did not consider the effect of nutritional status on hepatotoxicity. Second, community controls were enrolled in this study one to two months after their initial illness which precluded their weight measurements as it would have presumably increased. Third, the sample required for controls was not reached in either group due to constraints linked to restricted movement during the Ebola outbreak; therefore, we pooled both control groups to increase statistical power. Furthermore, the power of the study was calculated based the percentage of exposure to paracetamol rather than the dose itself. Paracetamol plasma levels from residual blood samples were also not available for controls. Fourth, case-definitions were not specific for paracetamol poisoning. We refined the case definition through syndromic surveillance, but multiple diagnoses among severely sick children make interpretation of case-ascertainment challenging, particularly in the case-control study.

## Conclusions

In a context with limited in diagnostic capacity, this study suggests that supratherapeutic doses of paracetamol should be considered as a primary cause of severe liver failure among children. In spite of the wide range of clinical presentations, our results are consistent with the possibility of paracetamol supratherapeutic overdose. These results are hypothesis-generating rather than confirmatory. Clinicians in similar contexts should include toxic paracetamol ingestions in their diagnosis of severely ill children. The lack of toxicological laboratory capacity in Liberia and other low-resource settings means that cases may not be recognized. Despite the importance of underdiagnoses, the clinical profile in most cases shows advanced stages of ALF where only supportive medical interventions are of limited effect. In this setting, we suggest that administration of N-acetylcysteine should be considered irrespective of plasma paracetamol concentrations. As such, preventive strategies and interventions, including education, are all the more important. Strategies in Monrovia targeting caregivers, formal and informal pharmacists, and healthcare providers simultaneously to increase awareness may help to mitigate the hazards of paracetamol supratherapeutic dosing.

## Data Availability

The datasets used and/or analysed during the current study are available from the corresponding author on request.

## Abbreviations

ALF: Acute liver failure
ALP: Alkaline Phosphatase
ALT: Alanine aminotransferase
AST: Aspartate aminotransferase
BJH: Bardnesville Junction Paediatric Hospital
CI: Confidence Interval
OR: Odds Ratio;
PEWS: Paediatric early warning score
RDT: Rapid Diagnostic Test
ULN: Upper limit of normal
