# Correction to: Suspected paracetamol overdose in Monrovia, Liberia: a matched case–control Study

**DOI:** 10.1186/s12887-020-02250-2

**Published:** 2020-08-14

**Authors:** Mohamad K. Haidar, Florian Vogt, Islam Amine Larabi, Kensuke Takahashi, Fanny Henaff, Lisa Umphrey, Nikola Morton, Luke Bawo, Joseph Kerkula, Robin Ferner, Klaudia Porten, Jean-Claude Alvarez, Frederic J. Baud

**Affiliations:** 1 Epicentre, 8 Rue Saint Sabin, 75011 Paris, France; 2 UMR 8257, Université Paris Descartes, Paris, France; 3 Department of Pharmacology and Toxicology, Paris Saclay University, Espace Technologique Bat. Discovery - RD 128 - 2e ét, 91190 Saint-Aubin, France; 4 Médecins sans Frontières – Operational Center Paris, Paris, France; 5 Ministry of Health and Social Welfare, Monrovia, Liberia; 6 Institute of Clinical Sciences, University of Birmingham, Birmingham, England; 7 Assistance Publique – Hôpitaux de Paris, Paris, France; 8 University Paris Diderot, Paris, France; 9 EA7323, Evaluation of prenatal and paediatric therapeutics and pharmacology, Université Paris Descartes, Paris, France

**Correction to: BMC Pediatr 20, 139 (2020)**

**https://doi.org/10.1186/s12887-020-2008-3**

Following the publication of the original article [1], there was error on the author group and the author contribution section. The authors Jean-Claude Alvarez and Islam Amine Larabi contributed to this manuscript by providing the analytical toxicology results. It was never our intention to exclude them as authors. Early on (in 2016) there were disagreements regarding authorship order for these two authors. Significant delays ensued and unfortunately the manuscript was submitted without ultimately adding their names to the list of authors. This inadvertent mistake was not realised during the proofing stage.

In the second part “Author Contribution”, we just added the contribution of the new authors to the Author Contribution statement (in bold below).

“MKH led the epidemiological and statistical analysis, drafted the manuscript, and interpretation of the results. FV and KT contributed towards designing the study and the data collection instruments. KP, FH, LU, and FJB contributed towards data analysis, interpretation of the results, and logistical support. REF and NM contributed substantially to the interpretation of the results. IAL and JCA contributed by providing the analytical toxicology results. LB and JK contributed to the conception and result interpretation.”

Therefore, the authors intend to correct the errors through publishing this correction paper.
